# A new AR therapy (MP29-02*): all of ARIA in one puff

**DOI:** 10.1186/2045-7022-5-S4-P42

**Published:** 2015-06-26

**Authors:** Ralph Mösges, Ludger Klimek, Carlos Baena-Cagnani, G Walter Canonica

**Affiliations:** 1 University of Cologne, IMSIE, Cologne, Germany; 2 Centre for Rhinology and Allergology, Allergy, Wiesbaden, Germany; 3 Catholic University of Cordoba, Respiratory Division, Cordoba, Argentina; 4 University of Genoa, IRCCS AOU, Genoa, Italy

## 

Allergic rhinitis (AR) is increasing in prevalence and becoming more difficult to treat. There is a subset of patients who are refractory to ARIA-defined rhinitis management approaches [1]. Even though new treatments have been made available for symptomatic relief, no new class of medication was forthcoming, until recently. The situation has now changed. MP29-02* is a novel intranasal formulation of azelastine hydrochloride (AZE) and fluticasone propionate (FP) in an advanced delivery system. It benefits from antihistamine, mast-cell stabilizing, anti-leukotriene and anti-inflammatory properties, made up in a unique formulation and delivered using an improved device (vs marketed intranasal steroid sprays (INS)). MP29-02*’s novel formulation and spray characteristics (e.g. finer droplet size, consistent spray release, wider spray angle) were developed to improve drug deposition on the nasal mucosa and ensure optimal retention. The impact has been observed both pharmacokinetically, [2] and clinically [3,4]. MP29-02* was created to be more effective than any existing symptomatic treatment for AR, have a rapid onset of action and a sustained effect. Delivered as a single spray from one device, the aim was to improve compliance, maximize convenience for patients and simplify dosing. This product provides more effective relief than currently considered gold standard treatment, INS. A recent publication by Meltzer et al, 4 re-assessed the efficacy of MP29-02* versus AZE and FP in an innovative and clinically relevant way by responder analyses. The authors determined different response cut-offs from 30 to 90% reflective total nasal symptom score (rTNSS) reduction from baseline. More MP29-02* patients achieved each response (vs FP or AZE), and days earlier. A response ceiling of ≥60% was identified above which INS failed to differentiate from placebo. This may explain why moderate/severe AR patients still complain of bothersome symptoms despite ARIA-guided treatment. Patients who remain symptomatic on monotherapy should experience a significant reduction in their symptoms with MP29-02*, exceeding that which they have experienced in the past, and many days faster than an INS. MP29-02* comprises all the pharmacological principles foreseen in the ARIA treatment algorithm.

*Dymista
